# 662. Impact of COVID-19 Mobile Vaccine Clinics on Health, Costs, and Equity: A Cost-Effectiveness Analysis in Utah

**DOI:** 10.1093/ofid/ofaf695.217

**Published:** 2026-01-11

**Authors:** Khanh Duong, Damon Toth, Danielle Nguyen, Yue Zhang, Richard Nelson, Andrew T Pavia, Barbara E Jones, Cindy Wynette, Makoto M Jones, Matthew H Samore, Nathorn Chaiyakunapruk

**Affiliations:** University of Utah, Salt Lake City, Utah; University of Utah, Salt Lake City, Utah; University of Utah, Salt Lake City, Utah; University of Utah, Salt Lake City, Utah; University of Utah, Salt Lake City, Utah; University of Utah, Salt Lake City, Utah; University of Utah and Salt Lake City VA Healthcare System, Salt Lake City, Utah; Utah Department of Health and Human Services, Salt Lake, Utah; Veterans Affairs, Salt Lake City, Utah; University of Utah, Salt Lake City, Utah; University of Utah, Salt Lake City, Utah

## Abstract

**Background:**

Mobile vaccine clinics (MVCs) were widely implemented in the United States (US) during the COVID-19 vaccination rollouts to overcome access barriers for underserved populations. However, the comprehensive value of these programs has not been fully assessed. This study aimed to estimate the health and economic outcomes of an MVC program that was deployed in the state of Utah beginning on April 1, 2021 across Hispanic and non-Hispanic populations in Utah.

**Figure:**
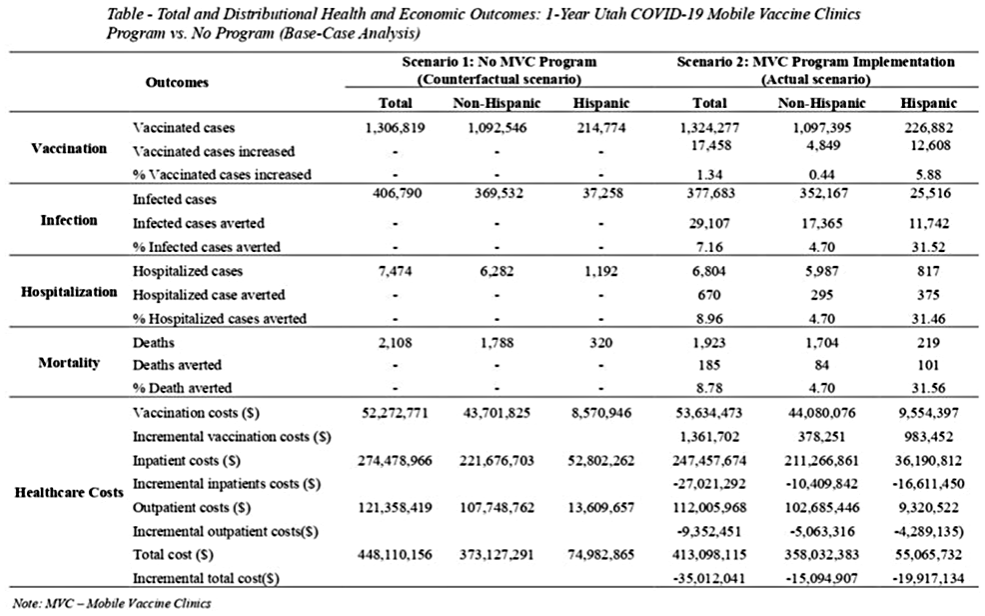

**Methods:**

We conducted a cost-effectiveness analysis over 1-year time horizon, comparing the MVC program implementation with a counterfactual scenario in which the MVC program was not implemented. The COVID-19 vaccinations in these scenarios were drawn from our previous estimates of MVC effect on vaccination uptake in Utah. The COVID-19 infections, hospitalizations, and deaths were projected using a Utah-calibrated Susceptible-Infected-Recovered (SIR) model. Direct healthcare costs and program costs were estimated from a healthcare system perspective in 2021 US dollars. Analyses were stratified by Hispanic and non-Hispanic populations.

**Results:**

In the base-case analysis, the one-year MVC program was estimated to increase 17,458 vaccines (1.34% relative increase compared to counterfactual scenario). This intervention averted an estimated 29,107 infections (7.16% reduction), 670 hospitalizations (8.96 % reduction), and 185 deaths (8.78% reduction) compared to the counterfactual scenario without the MVC program. The positive health impacts were disproportionately greater among Hispanic population compared to non-Hispanic population. The estimated cost of the MVC program implementation was $1,362,000. This investment was estimated to save $27,021,000 in averted inpatient costs and $9,352,000 in averted outpatient costs. Overall, the MVC program was estimated to save $35,012,000, representing a cost-saving intervention.

**Conclusion:**

The one-year MVC program in Utah improved health outcomes, saved costs, and enhanced equity. These findings support the use of MVCs as an effective, efficient, and equitable public health delivery strategy for immunizations and potentially other preventative services.

**Disclosures:**

Andrew T. Pavia, MD, Antimicrobial Therapy, Inc: Royalties for Sanford Guide

